# Efficacy and Safety of Blonanserin Oral Tablet in Adolescents with Schizophrenia: A 6-Week, Randomized Placebo-Controlled Study

**DOI:** 10.1089/cap.2021.0013

**Published:** 2022-02-14

**Authors:** Takuya Saito, Saori Sugimoto, Reiko Sakaguchi, Hiroshi Nakamura, Jun Ishigooka

**Affiliations:** ^1^ Department of Child and Adolescent Psychiatry, Hokkaido University Hospital, Sapporo, Japan.; ^2^ Sumitomo Dainippon Pharma Co., Ltd., Chuo-ku, Tokyo, Japan.; ^3^ Institute of CNS Pharmacology, Shibuya-ku, Tokyo, Japan.

**Keywords:** adolescent, antipsychotics, blonanserin, schizophrenia

## Abstract

***Objectives:*** To evaluate the short-term efficacy and safety of blonanserin in adolescents with schizophrenia.

***Methods:*** This 6-week multicenter, double-blind, randomized, placebo-controlled study investigated fixed-dose blonanserin (8 or 16 mg/day) in patients 12–18 years of age diagnosed with schizophrenia, as indicated by a Positive and Negative Syndrome Scale (PANSS) total score of 60–120 and a Clinical Global Impressions-Severity score of ≥3. The primary endpoint was change from baseline to week 6 in the PANSS total score, using a mixed model for repeated measures analysis. Safety was assessed by the incidence and severity of adverse events (AEs).

***Results:*** Among 151 randomized patients, 150 were included in the primary analysis population. Demographic and clinical characteristics were similar across groups at baseline. The rate of study discontinuation was 14.9%, 23.5%, and 28.3% in patients administered with placebo, blonanserin 8 mg/day, and blonanserin 16 mg/day, respectively. The least-squares mean change (95% confidence interval [CI]) from baseline to week 6 in PANSS total score was −10.6 (−16.10 to −5.10), −15.3 (−20.80 to −9.86), and −20.5 (−25.89 to −15.16) in patients administered placebo, 8 mg/day blonanserin, and 16 mg/day blonanserin, respectively. The 16-mg/day blonanserin group showed significantly greater reduction in the PANSS total score than the placebo group (least-squares mean difference [95% CI]: −9.9 [−17.61 to −2.25], *p* = 0.012, effect size: 0.538), although the 8-mg/day group showed no significant difference. The incidence of AEs such as akathisia, somnolence, and hyperprolactinemia was higher in the blonanserin groups than in the placebo group. AEs associated with blonanserin were generally mild and were consistent with its known profile in adults with schizophrenia.

***Conclusions:*** Blonanserin achieved a sufficient efficacy in adolescent patients, and the safety profile was similar to that in adults, which suggests that blonanserin may be a safe treatment option for adolescents with schizophrenia.

Study registration number: Japic CTI-111724.

## Introduction

Early-onset schizophrenia (schizophrenia occurring before the age of 18 years) has been reported to have a poor prognosis (Clemmensen et al. 2012; Immonen et al. 2017). Moreover, longer duration of untreated first-episode schizophrenia has been reported to lead to reduced social functioning (Penttilä et al. 2014; Díaz-Caneja et al. 2015). Therefore, particularly in adolescence, therapeutic intervention must be considered early and carefully.

Various guidelines for the treatment of schizophrenia recommend the combined use of pharmacological therapy (primarily antipsychotics) and psychosocial treatment (Hasan et al. 2012; McClellan et al. 2013; Abidi et al. 2017). In addition, the risk of relapse increases when patients with stable first-episode psychosis discontinue antipsychotics (Kishi et al. 2019); thus, these patients need antipsychotics that can be continued seamlessly from adolescence to adulthood. The “practice parameter for the assessment and treatment of children and adolescents with schizophrenia” developed by the American Academy of Child and Adolescent Psychiatry Council on November 2012 recommends the use of atypical antipsychotics as a clinical standard considering the balance between effectiveness and safety/tolerability as pharmacological therapies for schizophrenia spectrum disorders in children.

However, adolescent patients are particularly vulnerable to metabolic adverse reactions, and the use of drugs with a high risk for weight gain as first-line agents should be limited (McClellan et al. 2013). Therefore, paying attention to safety profiles is more important when selecting atypical antipsychotics, even if they have been approved for adolescent patients. In addition, some of the atypical antipsychotics used in adults have not been approved for use in adolescents and may be used in off-label ways.

The atypical antipsychotic blonanserin selectively binds to cerebral dopamine D_2_, D_3_, and serotonin 5-HT_2A_ receptors, acting as a full antagonist (Murasaki 2007; Harvey et al. 2019). In addition, blonanserin has been reported to sufficiently bind to cerebral dopamine D_3_ receptor similarly as cariprazine does (dopamine D_2_/D_3_ receptor partial agonist) (Sakayori et al. 2021), which can be a potential therapeutic target of action. In patients with adult schizophrenia, a randomized controlled study confirmed that blonanserin had greater efficacy than placebo in acute cases (Garcia et al. 2009). Other randomized controlled studies confirmed the noninferior efficacy of blonanserin to haloperidol (Murasaki 2007; Harvey et al. 2019), showing greater improvement in negative symptoms with fewer extrapyramidal adverse reactions than haloperidol as well as the noninferior efficacy of blonanserin to risperidone (Miura 2008; Harvey et al. 2020), with the following differences in safety profile: compared with risperidone, blonanserin was associated with a lower risk of blood prolactin increase, weight gain, and orthostatic hypotension; however, blonanserin was associated with a higher incidence of akathisia and excitability than risperidone.

Considering that efficacy for negative symptoms often leads to poor outcomes (Correll and Schooler 2020) and lower risk of metabolic adverse reactions associated with atypical antipsychotics, blonanserin can be a useful treatment option for adults with schizophrenia.

Blonanserin has not been approved for the treatment of adolescent patients with schizophrenia until recent approval in Japan. We report the results of a pivotal study for adolescents with schizophrenia and discuss the efficacy and safety of blonanserin treatment. This was the first multicenter, double-blind, randomized, placebo-controlled, parallel-group comparison study of oral blonanserin for adolescents with schizophrenia.

## Methods

### Patients

This study examined patients 12–18 years of age who were diagnosed with schizophrenia according to the Diagnostic and Statistical Manual of Mental Disorders, 4th Edition, Text Revision (DSM-IV-TR) and confirmed with the Mini-International Neuropsychiatric Interview for Children and Adolescents. The key inclusion criteria were a total score of 60–120 in the Positive and Negative Syndrome Scale (PANSS) and an assessment score of at least 3 (mildly ill) in the Clinical Global Impressions-Severity of Illness Scale (CGI-S).

The key exclusion criteria included the following: previous treatment with blonanserin; contraindications listed in the package insert for oral blonanserin; concurrent or previous malignant syndrome, tardive dyskinesia, paralytic ileus, rhabdomyolysis, agranulocytosis, pulmonary embolism, or deep vein thrombosis; Parkinson's disease; strong suicidal ideation and a history of suicide attempt or self-mutilation as a means of suicide; diabetes mellitus; complications such as serious cardiovascular, liver, kidney, organic brain, hematological, endocrinal, or spastic disease; a history of substance abuse or dependence and alcohol abuse or dependence; a history of clozapine treatment or psychiatric symptoms determined by the study investigator to have no improvement despite sufficient dose of at least two types of antipsychotics in the year (365 days) before screening; treatment with a depot antipsychotic preparation (sustained-release injection) in the 3 months (90 days) before screening; treatment with electroconvulsive therapy in the 6 months (180 days) before screening; and actual or possible pregnancy.

This study was approved in advance by the Institutional Review Boards of all the participating medical institutions and conducted in accordance with the ethical principles of the Declaration of Helsinki and in line with the regulatory requirements, including Japan's ministerial ordinance on Good Clinical Practice. After explaining all aspects of the study, written assent was obtained from all patients, and consent was obtained from their parents/legal guardians.

### Study design

**FIG. 1.**
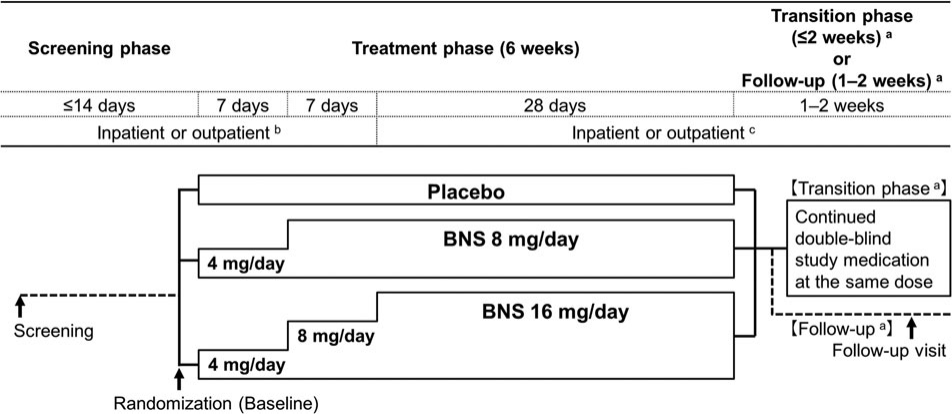
Study schematic. During the treatment phase, oral BNS tablet 8 or 16 mg/day, or placebo was administered twice daily, after the morning and evening meals, for 6 weeks. The same number of tablets were administered in each of the treatment groups using BNS tablets 2 mg, 4 mg, and identical placebo tablets. Concomitant antipsychotics, antiparkinson drugs, psychotropics, hypnotics, and psychotherapeutic interventions were restricted from screening to follow-up in accordance with the study protocol. ^a^Transition phase, for patients who continued in long-term extension study; Follow-up, for patients who did not enter in long-term extension study. ^b^Patients with a CGI-SS item 1 score of 1 (not at all suicidal) at screening who met other specific criteria were allowed to be shifted to outpatient observation during the study period at the physicians' discretion. c: Patients with a CGI-SS item 1 score ≥2 at screening were hospitalized until observation and assessment after 2 weeks were completed. Subsequently, if patients or their legal representatives requested and they met other specific criteria, they were allowed to be switched to outpatient observation. BNS, blonanserin; CGI-SS, Clinical Global Impressions of Suicide Severity.

This multicenter randomized, double-blind, placebo-controlled, parallel-group comparison study was conducted from March 2012 to March 2019 at 73 medical institutions in Japan. The study comprised a screening phase and a 6-week treatment phase. Study patients were randomly allocated at a 1:1:1 ratio into the placebo, 8-mg/day blonanserin, and 16-mg/day blonanserin groups using computer-generated randomization. Oral tablets were administered twice daily, after the morning and evening meals, for 6 weeks in a double-blind manner (Fig. 1).

The investigator responsible for the study drug allocation prepared and appropriately stored a study drug allocation table. After the patients were enrolled, the study sites used the allocated study drug. The investigator responsible for the study drug allocation prepared an emergency key held by the study sponsor to unlock the data in case of an emergency. The institution performing drug concentration measurement submitted a report of its results after unlocking the data with the key.

The blonanserin dose was 8 mg/day, the lower limit of the adult maintenance dose, or 16 mg/day, the upper limit. Considering adolescent safety, dosing was started at 4 mg/day, half the lower limit of the adult maintenance dose. A double dummy method was used, with each group receiving the same set number of tablets as follows: 1 tablet for each dose from day 1 to 14 and 2 tablets for each dose starting from day 15. The patients allocated to the 8-mg/day blonanserin group started receiving a dose of 4 mg/day, which was increased to 8 mg/day after 1 week. The starting dose for the patients allocated to the 16-mg/day blonanserin group was 4 mg/day and was increased to 8 mg/day after 1 week and 16 mg/day after 2 weeks (Fig. 1). The treatment adherence was confirmed on the basis of the number of tablets returned at study visits.

Patients whose suicide severity was ≥2 as assessed by the Clinical Global Impressions of Suicide Severity (CGI-SS) item 1 at screening were hospitalized from screening until the week 2 observation, and the assessment was completed. Subsequently, upon request from the patients and their parents/legal guardians, switching to outpatient-basis study participation was allowed if the following criteria were met: sufficient support from caregivers for outpatient treatment and routine outpatient visits, no clinical change in suicidal risk (e.g., no CGI-SS score changes and no suicide-related adverse event [AE]), and stable psychiatric symptoms on PANSS or CGI-S scores (e.g., CGI-S assessment score of ≤4).

Patients with CGI-SS item 1 score of 1 at screening (not at all suicidal) were allowed to participate in the study on an outpatient basis if the following criteria were met: cohabitation with the parents/legal guardians, immediate contact with the study investigator is possible in case of emergency, and CGI-S assessment score of ≤4 at screening (moderately ill; Fig. 1). However, if suicidal risk of a patient increased beyond acceptable level during outpatient observation, the patient could be hospitalized at the study site if the physician determined that withdrawal was not warranted. If hospitalization was not possible, the patient was withdrawn from the study.

### Concomitant medications

Concomitant use of antipsychotics other than the study drug was prohibited. Concomitant use of psychotropics and hypnotics was allowed as needed but limited to only one of the following drugs: among psychotropics, etizolam, flutazolam, clotiazepam, or lorazepam was allowed and among hypnotics, brotizolam, triazolam, lormetazepam, zopiclone, rilmazafone, or eszopiclone was allowed. The use of the drugs was prohibited within 12 hours before the efficacy assessments. Regarding the concomitant use of antiparkinsonian agents, biperiden (≤6 mg/day) was allowed if extrapyramidal symptoms newly occurred or worsened. Drug treatment for concurrent diseases (e.g., hypertension and dyslipidemia) was continued without changing the dosage and administration unless the concurrent disease worsened or improved.

The use of antimanic or antiepileptic agents, monoamine oxidase inhibitors, CYP3A4 inhibitors (except for topical external agents), epinephrine (except when used as emergency intervention for anaphylaxis), and other study drugs or postmarketing clinical study drugs was prohibited. Changing or starting psychotherapy was prohibited, except when a patient was hospitalized or discharged. The use of electroconvulsive therapy was prohibited.

### Efficacy assessments

Efficacy was assessed at baseline and at weeks 1, 2, 4, and 6 using PANSS, CGI-S, and CGI-Improvement (CGI-I). The primary efficacy endpoint was the change in PANSS total score from baseline (before randomization at the start of treatment) to week 6 (Kay et al. 1987). Secondary endpoints were the change in PANSS total score at each assessment point; change in PANSS subscale scores at the last observation carried forward (LOCF) endpoint and each assessment point (Perkins et al. 2000); change in PANSS 5-factor model scores (Lindenmayer et al. 1994); change in each PANSS symptom score; PANSS remission rate (Andreasen et al. 2005); percentage of PANSS responders (Leucht et al. 2017); change in CGI-S score (Guy 1976); rate of improvement in CGI-I (percentage of patients with a CGI-I score of 1 [very much improved] or 2 [much improved]); and days from start to last date of administration. PANSS assessments were performed by the assessors who were trained and certified.

### Pharmacokinetic assessment

The plasma concentration of unchanged blonanserin, the main active moiety, was measured. Samples for pharmacokinetic assessment were collected at weeks 2 and 6.

### Safety assessments

Safety was assessed by examining the incidence and severity of AEs. AEs were recorded and classified using version 21.1 of the Medical Dictionary for Regulatory Activities of the International Council for Harmonization of Technical Requirements for Pharmaceuticals for Human Use. Extrapyramidal symptoms were evaluated by examining change in total score on the Drug-Induced Extrapyramidal Symptoms Scale (DIEPSS) (Inada et al. 2003) and percentage of patients using antiparkinsonian agents at last assessment and at each assessment point. DIEPSS is a physician-rating scale to assess the severity of extrapyramidal symptoms induced by antipsychotics on a 5-rank scale of 0 (normal) to 4 (severe) for each of the 8-symptom categories (gait, bradykinesia, sialorrhea, muscle rigidity, tremor, akathisia, dystonia, and dyskinesia) and one global assessment (overall severity). Suicide risk was evaluated by examining the change in CGI-SS score and deterioration rate (Meltzer et al. 2003) at week 6 (LOCF) and at each assessment point. CGI-SS is an overall clinician-rating scale of the clinical risk of suicidality and change in suicidality.

The CGI-SS item 1 has five severity levels of suicidality in the past 7 days: 1, not at all suicidal; 2, mildly suicidal; 3, moderately suicidal; 4, severely suicidal; and 5, attempted suicide. The CGI-SS item 2 has seven change levels from baseline in suicidality: 1, very much improved; 2, much improved; 3, minimally improved; 4, no change; 5, minimally worsened; 6, much worse; and 7, very much worse. Changes in laboratory test values, vital signs, and body weight as well as electrocardiographic findings and parameters were evaluated at the last testing point and each testing point.

### Statistical analysis

The SAS version 9.4 software was used to perform statistical analysis. Sample size was calculated to detect statistical difference for the primary endpoint using two-step closed testing procedure at one-sided significance level of 0.025 and power of 80%. On the basis of the results of placebo-controlled studies of other second-generation antipsychotic agents in patients with adolescent schizophrenia (Mathis 2007; Findling et al. 2008; Astra Zeneca 2009; Kryzhanovskaya et al. 2009), a difference in the change in PANSS total score of 10–13 and SD of 17–20 was assumed between the placebo group and 8-mg/day and 16-mg/day blonanserin groups. Consequently, 40–48 patients were required per group. Including patients excluded from the analysis, the target number of patients was set at 50 per group (a total of 150 in three groups).

The full analysis set (FAS) was used as the primary efficacy analysis set. The FAS comprised enrolled patients diagnosed with schizophrenia according to DSM-IV-TR who received the study drug at least once and had both baseline assessment and at least one postbaseline assessment of PANSS total score. The safety analysis set comprised patients who received the study drug at least once.

The primary endpoint was analyzed using a mixed model for repeated measures (MMRM) with the treatment groups as the fixed effect, and each assessment point, PANSS total score at baseline, and the interaction of the treatment group and assessment point as covariates. A closed testing procedure was used in the order of step 1 and step 2 to adjust for test multiplicity in the analysis of the primary endpoint. In step 1, contrast coefficients (placebo, 8-mg/day blonanserin, and 16-mg/day blonanserin groups) were set as −2, 1, and 1, respectively. If a significant difference in efficacy was observed between the active drug group (i.e., combined 8- and 16-mg/day blonanserin group) and placebo group in a two-sided test at the significant level of 0.05, the analysis proceeded to step 2, in which a two-sided test at the significance level of 0.05 was performed on the superiority of each dose group over the placebo group.

Regarding secondary endpoints, multiplicity of statistical tests was not adjusted. CGI-S changes were analyzed using MMRM. PANSS remission rate, percentage of PANSS responders, and rate of improvement in CGI-I were analyzed using Chi-square test or Fisher's exact test. *Posthoc* analysis was conducted for PANSS subscale scores and PANSS 5-factor model scores using MMRM in the same manner of statistical modeling as the primary endpoint.

## Results

### Patient disposition and baseline characteristics

**FIG. 2.**
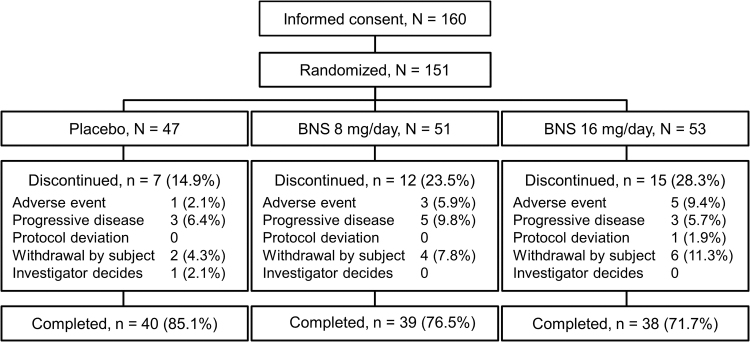
Patient disposition. One patient in the BNS 16-mg/day group without baseline PANSS total score was excluded from the FAS. Consequently, the number of patients in the FAS for placebo, BNS 8-mg/day, and16-mg/day groups were 47, 51, 52, respectively. BNS, blonanserin; PANSS, Positive and Negative Syndrome Scale; FAS, full analysis set.

Consent was obtained from 160 individuals, of whom 151 met all the inclusion criteria and were randomized as follows for double-blind treatment: 47 patients in the placebo, 51 patients in the 8-mg/day blonanserin, and 53 patients in the 16-mg/day blonanserin groups. The FAS comprised 150 patients, with the exclusion of 1 patient from the 16-mg/day blonanserin group who had no baseline PANSS total score (Fig. 2). Baseline characteristics of patients included in the FAS are summarized in Table 1.

**Table 1.** Demographic and Clinical Characteristics in Patients at Baseline (Full Analysis Set)

	Placebo (*N* = 47)	Blonanserin (8 mg/day) (*N* = 51)	Blonanserin (16 mg/day) (*N* = 52)
Sex, male, *n* (%)	20 (42.6)	21 (41.2)	23 (44.2)
Age (years), mean (SD)	15.6 (1.69)	15.3 (1.49)	15.6 (1.68)
Age (years), ≥15, *n* (%)	33 (70.2)	35 (68.6)	36 (69.2)
Height (cm), mean (SD)	161.09 (7.293)	160.89 (6.928)	162.64 (8.247)
Weight (kg), mean (SD)	52.36 (7.943)	56.47 (11.951)	57.06 (11.671)
Weight (kg), ≥50 kg, *n* (%)	30 (63.8)	34 (66.7)	36 (69.2)
BMI (kg/m^2^), mean (SD)	20.14 (2.391)	21.74 (3.987)	21.54 (3.937)
DSM-IV subtype, *n* (%)			
Disorganized	13 (27.7)	3 (5.9)	9 (17.3)
Catatonic	3 (6.4)	5 (9.8)	7 (13.5)
Paranoid	17 (36.2)	26 (51.0)	20 (38.5)
Residual	0	1 (2.0)	1 (1.9)
Undifferentiated	14 (29.8)	16 (31.4)	15 (28.8)
No. of episodes, *n* (%)			
1	38 (80.9)	42 (82.4)	36 (69.2)
≥2	7 (14.9)	8 (15.7)	16 (30.8)
Unknown	2 (4.3)	1 (2.0)	0
Hospitalization, *n* (%)			
Inpatient	22 (46.8)	29 (56.9)	27 (51.9)
Outpatient	25 (53.2)	22 (43.1)	25 (48.1)
Age at initial diagnosis (years), mean (SD)	13.4 (2.21)	13.3 (2.00)	13.1 (2.20)
Duration of illness (years), mean (SD)	2.13 (1.656)	1.98 (1.427)	2.53 (2.170)
Duration of current episodes (days), mean (SD)	635.2 (646.32)	540.8 (493.97)	602.0 (609.13)
Baseline PANSS total score, mean (SD)	89.8 (10.41)	86.5 (13.53)	88.7 (13.81)
PANSS composite subscale, *n* (%)			
Positive subscale score > Negative subscale score	15 (31.9)	21 (41.2)	16 (30.8)
Positive subscale score = Negative subscale score	3 (6.4)	2 (3.9)	2 (3.8)
Positive subscale score < Negative subscale score	29 (61.7)	28 (54.9)	34 (65.4)
Baseline CGI-S score, mean (SD)	3.98 (0.642)	3.94 (0.785)	3.98 (0.542)
Baseline CGI-SS score, mean (SD)	1.13 (0.337)	1.06 (0.238)	1.08 (0.269)
Baseline DIEPSS total score, mean (SD)	0.47 (1.300)	0.71 (2.773)	0.56 (1.662)

BMI, body mass index; CGI-S, Clinical Global Impressions-Severity; CGI-SS, Clinical Global Impressions of Severity of Suicidality; DIEPSS, Drug-Induced Extrapyramidal Symptoms Scale; DSM-IV, Diagnostic and Statistical Manual of Mental Disorders, 4th edition; PANSS, Positive and Negative Syndrome Scale; SD, standard deviation.

The discontinuation rate was 14.9% in the placebo, 23.5% in the 8-mg/day blonanserin, and 28.3% in the 16-mg/day blonanserin groups. The common reasons for discontinuation were progressive disease, AE, and withdrawal by subject (Fig. 2). The mean adherence rate exceeded 96% in each group, with a mean dosing duration of ≥35 days in all groups. Concomitant medications used during the treatment phase in the placebo, 8-mg/day blonanserin, and 16-mg/day blonanserin groups were psychotropics in 44.7%, 62.7%, and 55.8%, respectively, and hypnotics in 48.9%, 68.6%, and 61.5%, respectively.

### Efficacy

**FIG. 3.**
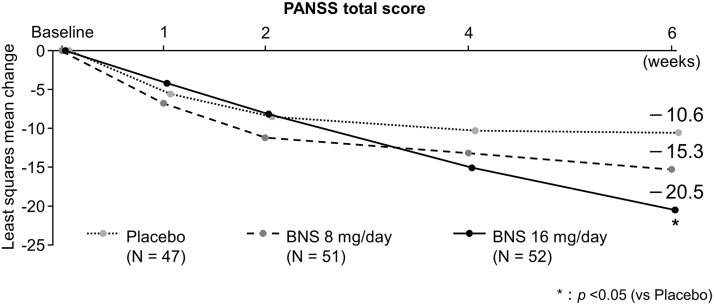
Change from baseline in PANSS total score (MMRM). Coefficient of contrast to test global null hypothesis is (Placebo, BNS 8 mg/day, 16 mg/day) = (−2, 1, 1). Closed Testing Procedure was applied to adjust multiplicity. Only when global null hypothesis was rejected, BNS8- or 16-mg/day group was compared with Placebo group with 0.05 as significance level. BNS, blonanserin; MMRM, mixed model for repeated measures; PANSS, positive and negative syndrome scale.

The PANSS total score decreased from week 1 in all groups. The mean change in PANSS total score from baseline at week 6 (least square [LS] mean [95% confidence interval; CI]) was −10.6 (−16.10 to −5.10) in the placebo, −15.3 (−20.80 to −9.86) in the 8-mg/day blonanserin, and −20.5 (−25.89 to −15.16) in the 16-mg/day blonanserin groups. A significantly larger decrease in PANSS total score was observed in the 16-mg/day blonanserin group than in the placebo group (LS mean difference [95% CI]: −9.9 [−17.61 to −2.25], *p* = 0.012, effect size [ES]: 0.538); whereas no significant difference in change was observed in the 8-mg/day blonanserin group (LS mean difference [95% CI]: −4.7 [−12.49 to 3.03], *p* = 0.230, ES: 0.256) compared with the placebo group (Fig. 3; Table 2).

**Table 2.** Change from Baseline in Positive and Negative Syndrome Scale Total and Clinical Global Impression-Severity of Illness Scale Scores

	Placebo (*N* = 47)		Blonanserin (8 mg/day) (*N* = 51)			Blonanserin (16 mg/day) (*N* = 52)		
	Baseline, mean (SD)	Change at week 6, LS mean (95% CI)	Baseline, mean (SD)	Change at week 6, LS mean (95% CI)	Treatment difference,* p*-value	Baseline, mean (SD)	Change at week 6, LS mean (95% CI)	Treatment difference,* p*-value
PANSS total	89.8 (10.41)	−10.6 (−16.10 to −5.10)	86.5 (13.53)	−15.3 (−20.80 to −9.86)	0.230	88.7 (13.81)	−20.5 (−25.89 to −15.16)	0.012
CGI-S	3.98 (0.642)	−0.45 (−0.775 to −0.128)	3.94 (0.785)	−0.83 (−1.149 to −0.502)	0.108	3.98 (0.542)	−0.84 (−1.158 to −0.522)	0.092

Estimates, CIs, and *p*-values are based on a MMRM of the change from baseline score, with fixed effects for treatment group, visit as a categorical variable, baseline score, and treatment by visit interaction. An UN is used for within-patient covariance matrix. For models that failed to converge under an UN, a different covariance matrix was used instead: heterogeneous Toeplitz (TOEPH), heterogeneous first-order autoregressive (ARH(1)), or Toeplitz (TOEP). These alternative covariance structures used a robust sandwich estimator for the standard error of the fixed effects estimate. In case that all models failed to converge, ‘Noncalculable’ was presented instead of model estimates. Coefficient of contrast to test global null hypothesis was (Placebo, Blonanserin 8-mg/day, 16-mg/day) = (−2, 1, 1). Closed Testing Procedure was applied to adjust multiplicity. Only when global null hypothesis was rejected, Blonanserin 8- or 16-mg/day group was compared with Placebo group with 0.05 as significance level. *p*-Value: Blonanserin 8- or 16-mg/day group was compared with placebo group, and the multiplicity was not adjusted except for the primary endpoint.

CI, confidence interval; CGI-S, Clinical Global Impressions-Severity; LS mean, least squared mean; MMRM, mixed model for repeated measures; PANSS, Positive and Negative Syndrome Scale; SD, standard deviation; UN, unstructured matrix.

The mean change from baseline in CGI-S score (LS mean [95% CI]) at week 6 was −0.45 (−0.775 to −0.128) in the placebo, −0.83 (−1.149 to −0.502) in the 8-mg/day blonanserin, and −0.84 (−1.158 to −0.522) in the 16-mg/day blonanserin groups, with a difference from the placebo group of −0.37 (−0.832 to 0.083) in the 8-mg/day blonanserin group and −0.39 (−0.843 to 0.065) in the 16-mg/day blonanserin group (*p* = 0.108 for the 8-mg/day blonanserin group and *p* = 0.092 for the 16-mg/day blonanserin group; MMRM; Table 2).

The mean changes in PANSS subscale scores and PANSS total 5-factor model scores were largest in the 16-mg/day blonanserin group, followed by the 8-mg/day blonanserin and placebo groups (Table 3 and Table 4).

**Table 3.** Change from Baseline in Positive and Negative Syndrome Scale Subscale and Positive and Negative Syndrome Scale Five-Factor Model Scores (Planned Analysis)

	Placebo (*N* = 47)		Blonanserin (8 mg/day) (*N* = 51)		Blonanserin (16 mg/day) (*N* = 52)	
	Baseline, mean (SD)	Change at Week 6 (LOCF), mean (SD)	Baseline, mean (SD)	Change at week 6 (LOCF), mean (SD)	Baseline, mean (SD)	Change at week 6 (LOCF), mean (SD)
PANSS subscale						
Positive	19.9 (3.14)	−2.1 (5.83)	20.4 (4.69)	−3.6 (6.23)	20.1 (3.23)	−4.6 (5.27)
Negative	23.3 (4.51)	−2.7 (5.39)	21.9 (4.75)	−3.4 (4.51)	22.6 (5.62)	−4.6 (4.70)
General psychopathology	46.6 (6.02)	−5.6 (12.45)	44.3 (7.45)	−6.8 (8.60)	45.9 (7.91)	−8.9 (10.19)
PANSS five factor						
Negative symptoms score	20.9 (4.87)	−2.6 (4.63)	19.3 (4.46)	−3.5 (4.44)	20.0 (5.31)	−4.4 (5.05)
Excitement score	10.3 (2.29)	−0.7 (3.64)	10.5 (2.47)	−1.5 (3.24)	10.2 (2.35)	−1.6 (3.04)
Cognitive disorders score	13.7 (2.91)	−1.3 (4.03)	13.7 (3.36)	−1.9 (3.38)	14.2 (3.07)	−2.7 (2.61)
Positive symptoms score	12.1 (2.19)	−1.7 (3.76)	11.9 (2.89)	−2.3 (3.67)	11.8 (2.64)	−3.2 (3.30)
Anxiety/depression score	14.7 (2.78)	−1.9 (4.05)	14.1 (2.80)	−2.3 (3.07)	14.3 (3.37)	−2.8 (4.14)

Week 6 (LOCF) is the last postbaseline observation in the treatment phase up to 7 days after final date of the study drug administration, except for transition phase. Only data from patients with both baseline and the corresponding visit values available were used to compute statistical summaries.

LOCF, last observation carried forward; PANSS, Positive and Negative Syndrome Scale; SD, standard deviation.

**Table 4.** Change from Baseline in Positive and Negative Syndrome Scale Subscale and Positive and Negative Syndrome Scale Five-Factor Model Scores (*Posthoc* Analysis by Mixed Model for Repeated Measures)

	Placebo (*N* = 47)		Blonanserin (8 mg/day) (*N* = 51)			Blonanserin (16 mg/day) (*N* = 52)		
	Baseline, mean (SD)	Change at week 6, LS mean (95% CI)	Baseline, mean (SD)	Change at week 6, LS mean (95% CI)	Treatment difference,* p*-value	Baseline, mean (SD)	Change at week 6, LS mean (95% CI)	Treatment difference,* p*-value
PANSS subscale								
Positive	19.9 (3.14)	−2.3 (−3.88 to −0.78)	20.4 (4.69)	−4.0 (−5.54 to −2.45)	0.135	20.1 (3.23)	−5.3 (−6.86 to −3.83)	0.007
Negative	23.3 (4.51)	−2.8 (−4.20 to −1.42)	21.9 (4.75)	−3.9 (−5.25 to −2.48)	0.290	22.6 (5.62)	−5.2 (−6.60 to −3.87)	0.015
General psychopathology	46.6 (6.02)	−5.8 (−8.67 to −2.95)	44.3 (7.45)	−7.8 (−10.62 to −4.93)	0.336	45.9 (7.91)	−10.2 (−13.00 to −7.41)	0.031
PANSS five-factor								
Negative symptoms score	20.9 (4.87)	−2.6 (−3.88 to −1.24)	19.3 (4.46)	−4.0 (−5.27 to −2.65)	0.139	20.0 (5.31)	−5.1 (−6.37 to −3.78)	0.008
Excitement score	10.3 (2.29)	−0.9 (−1.78 to −0.09)	10.5 (2.47)	−1.7 (−2.51 to −0.82)	0.228	10.2 (2.35)	−2.0 (−2.84 to −1.17)	0.076
Cognitive disorders score	13.7 (2.91)	−1.4 (−2.32 to −0.40)	13.7 (3.36)	−2.1 (−3.09 to −1.18)	0.258	14.2 (3.07)	−3.0 (−3.96 to −2.08)	0.016
Positive symptoms score	12.1 (2.19)	−1.8 (−2.74 to −0.84)	11.9 (2.89)	−2.7 (−3.63 to −1.73)	0.195	11.8 (2.64)	−3.7 (−4.65 to −2.78)	0.005
Anxiety/depression score	14.7 (2.78)	−1.9 (−2.91 to −0.99)	14.1 (2.80)	−2.7 (−3.61 to −1.69)	0.305	14.3 (3.37)	−3.3 (−4.23 to −2.34)	0.051

Estimates, CIs, and *p*-values are based on a MMRM of the change from baseline score, with fixed effects for treatment group, visit as a categorical variable, baseline score, and treatment by visit interaction. An UN is used for within-patient covariance matrix. For models that failed to converge under an UN, a different covariance matrix was used instead: heterogeneous Toeplitz (TOEPH), heterogeneous first-order autoregressive (ARH(1)), or Toeplitz (TOEP). These alternative covariance structures used a robust sandwich estimator for the standard error of the fixed effects estimate. In case that all models failed to converge, “Noncalculable” was presented instead of model estimates. *p*-Value: Blonanserin 8- or 16-mg/day group was compared with placebo group, and the multiplicity was not adjusted.

CI, confidence interval; LS mean, least squared mean; MMRM, mixed model for repeated measures; PANSS, Positive and Negative Syndrome Scale; SD, standard deviation; UN, unstructured matrix.

The PANSS remission rate at week 6 (LOCF) was highest in the 16-mg/day blonanserin group, followed by the 8-mg/day blonanserin and placebo groups (Table 5). The percentage of PANSS responders at week 6 (LOCF) was largest in the 16-mg/day blonanserin group for all improvement criteria, followed by the 8-mg/day blonanserin and placebo groups. A statistically significant difference was observed between the 16-mg/day blonanserin and the placebo groups at an improvement criterion of 30%–50% or higher (Table 5). The rate of improvement in CGI-I at week 6 (LOCF) was 17.4% in the placebo, 35.3% in the 8-mg/day blonanserin, and 42.3% in the 16-mg/day blonanserin groups, which was higher in the 16-mg/day blonanserin group than in the placebo group (*p* = 0.009; Table 5).

**Table 5.** Analysis of Responder Rate in Positive and Negative Syndrome Scale Total Score, Remission Rate in Positive and Negative Syndrome Scale Total Score, and Improvement Rate in CGI-Improvement Score

	Placebo (*N* = 47)		Blonanserin (8 mg/day) (*N* = 51)			Blonanserin (16 mg/day) (*N* = 52)		
	Baseline,* n *(%)	Week 6 (LOCF),* n *(%)	Baseline,* n *(%)	Week 6 (LOCF),* n *(%)	Treatment difference,* p*-value	Baseline,* n *(%)	Week 6 (LOCF),* n *(%)	Treatment difference,* p*-value
PANSS responder								
≥20% improvement		25 (53.2)		28 (54.9)	0.817		34 (65.4)	0.210
≥30% improvement		15 (31.9)		21 (41.2)	0.323		31 (59.6)	0.006
≥40% improvement		9 (19.1)		13 (25.5)	0.376		25 (48.1)	0.003
≥50% improvement		6 (12.8)		11 (21.6)	0.223		16 (30.8)	0.034
PANSS remission rate	4 (8.5)	21 (44.7)	8 (15.7)	25 (49.0)	0.690	7 (13.5)	32 (61.5)	0.109
CGI-I rate		8 (17.4)		18 (35.3)	0.066		22 (42.3)	0.009

Week 6 (LOCF) is the last postbaseline observation in the treatment phase up to 7 days after final date of the study drug administration, except for transition phase. PANSS responders are defined as patients with an improvement in PANSS total score from baseline value, where lower observed PANSS total scores indicate lower severity of schizophrenia. Responders are assessed at levels of improvement from baseline ≥20%, 30%, 40%, and 50%. *p*-Value: Blonanserin 8- or 16-mg/day group was compared with placebo group by chi-square test, and the multiplicity was not adjusted. PANSS remission rate is defined as the proportion of patients with grade 3 (mild) or less in all the following items: delusions (P1), conceptual disorganization (P2), hallucinatory behavior (P3), blunted affect (N1), passive/apathetic social withdrawal (N4), lack of spontaneity and flow of conversation (N6), mannerisms and posturing (G5), and unusual thought content (G9). *p*-Value: Blonanserin 8- or 16-mg/day group was compared with placebo group by Fisher's exact test, and the multiplicity was not adjusted. CGI-I is defined as score of “very much improved” or “much improved” (CGI-I score of 1 or 2). *p*-Value: Blonanserin 8- or 16-mg/day group was compared with placebo group by Fisher's exact test and the multiplicity was not adjusted.

CGI-I, CGI-Improvement; LOCF, last observation carried forward; PANSS, Positive and Negative Syndrome Scale.

### Pharmacokinetics

At week 6, the approximate trough plasma blonanserin concentration (blood drawn >10 hours after study drug administration; mean [SD]) was 0.25 (0.12) ng/mL in the 8-mg/day blonanserin group (*n* = 38) and 0.45 (0.19) ng/mL in the 16-mg/day blonanserin group (*n* = 36).

### Safety

Safety was assessed in the 151 patients who received the study drug at least once. The incidence of AEs during the treatment phase was 68.1% in the placebo, 80.4% in the 8-mg/day blonanserin, and 92.5% in the 16-mg/day blonanserin groups. Most AEs were mild or moderate in all the groups. Severe AEs included worsening schizophrenia (two patients in the placebo group, one in the 8-mg/day blonanserin, and one in the 16-mg/day blonanserin) and somnolence (one patient in the 16-mg/day blonanserin group). A causal relationship with the study drug could not be ruled out for worsening schizophrenia in two patients from the placebo group and for somnolence. Serious AEs included worsening schizophrenia (one patient in the placebo group) and hyperventilation (one patient in the 8-mg/day blonanserin group). A causal relationship with the study drug could not be ruled out for either of the serious AEs. None of the patients died. AEs led to the discontinuation of administration in three patients in the placebo group, five in the 8-mg/day blonanserin group, and six in the 16-mg/day blonanserin group (Table 6).

**Table 6.** Summary of Adverse Events

	Placebo (*N* = 47)	Blonanserin (8 mg/day) (*N* = 51)	Blonanserin (16 mg/day) (*N* = 53)
AEs	32 (68.1)	41 (80.4)	49 (92.5)
Mild	26 (55.3)	33 (64.7)	31 (58.5)
Moderate	4 (8.5)	7 (13.7)	16 (30.2)
Severe	2 (4.3)	1 (2.0)	2 (3.8)
Somnolence	0	0	1 (1.9)
Schizophrenia	2 (4.3)	1 (2.0)	1 (1.9)
Serious	1 (2.1)	1 (2.0)	0
Schizophrenia	1 (2.1)	0	0
Hyperventilation	0	1 (2.0)	0
Death	0	0	0
AE leading to discontinuation of study drug^a^	3 (6.4)	5 (9.8)	6 (11.3)
Nausea	1 (2.1)	0	1 (1.9)
Vomiting	0	0	1 (1.9)
Malaise	0	0	1 (1.9)
Hepatic function abnormal	0	1 (2.0)	0
Gastroenteritis	0	1 (2.0)	0
Blood prolactin increase	0	0	1 (1.9)
Akathisia	0	0	1 (1.9)
Somnolence	0	1 (2.0)	2 (3.8)
Headache	1 (2.1)	0	0
Schizophrenia	2 (4.3)	2 (3.9)	1 (1.9)
AE related to Extrapyramidal syndrome^b^	2 (4.3)	13 (25.5)	27 (50.9)
AE related to Prolactin increase^c^	2 (4.3)	8 (15.7)	17 (32.1)
Weight increased	0	2 (3.9)	0
Weight decreased	1 (2.1)	0	0

All AEs were coded using MedDRA dictionary version 21.1.

^a^ A patient may have had two or more AEs, thus the same patient may appear in different AEs in the following breakdown.

^b^ Patients with any Extrapyramidal syndrome AE such as oculogyric crisis, salivary hypersecretion, muscle rigidity, akathisia, tremor, dystonia, dyskinesia, bradykinesia, extrapyramidal disorder, myoclonus, or parkinsonian gait.

^c^ Patients with any Prolactin increased AE such as hyperprolactinemia, blood prolactin increased, or galactorrhea.

AE, adverse event.

The AEs that occurred in ≥5% of the patients in the pooled blonanserin group are shown in Table 7. The incidence rates of akathisia, somnolence, hyperprolactinemia, increase in blood prolactin, tremor, and dystonia were relatively high in the pooled blonanserin group compared with those in the placebo group. There was no clinically relevant difference in the incidence of headache, nausea, worsening schizophrenia, or skin abrasion between the pooled blonanserin and placebo groups.

**Table 7.** Common Adverse Events (≥ 5% Incidence in the Pooled Blonanserin Group)

System organ class	Placebo (*N* = 47)	Blonanserin (8 mg/day) (*N* = 51)	Blonanserin (16 mg/day) (*N* = 53)
Preferred term,* n *(%)			
Endocrine disorders			
Hyperprolactinemia	1 (2.1)	5 (9.8)	9 (17.0)
Gastrointestinal disorders			
Nausea	4 (8.5)	5 (9.8)	4 (7.5)
Injury, poisoning, and procedural complications			
Skin abrasion	3 (6.4)	2 (3.9)	4 (7.5)
Investigations			
Blood prolactin increase	1 (2.1)	3 (5.9)	7 (13.2)
Nervous system disorders			
Akathisia	2 (4.3)	7 (13.7)	17 (32.1)
Somnolence	1 (2.1)	8 (15.7)	10 (18.9)
Headache	6 (12.8)	6 (11.8)	4 (7.5)
Tremor	0	5 (9.8)	5 (9.4)
Dystonia	0	1 (2.0)	7 (13.2)
Psychiatric disorders			
Schizophrenia	3 (6.4)	4 (7.8)	2 (3.8)

All AEs were coded using MedDRA dictionary version 21.1.

AE, adverse event.

The incidence of AEs related to extrapyramidal symptoms was 4.3% in the placebo group, 25.5% in the 8-mg/day blonanserin group, and 50.9% in the 16-mg/day blonanserin group (Table 6). The mean change (SD) from baseline in total DIEPSS score at week 6 (LOCF) was −0.04 (0.833) in the placebo, 0.08 (1.560) in the 8-mg/day blonanserin, and 0.67 (2.324) in the 16-mg/day blonanserin groups. The percentage of patients who used antiparkinsonian agents was 4.3% in the placebo, 21.6% in the 8-mg/day blonanserin, and 34.0% in the 16-mg/day blonanserin groups. The rate of deterioration in CGI-SS item 2 score (percentage of 6 [much worse] or 7 [very much worse]) at week 6 (LOCF) was 4.3% in the placebo, 0% in the 8-mg/day blonanserin, and 1.9% in the 16-mg/day blonanserin groups, without clear increase in suicide risk. The serum prolactin level in the female patients (mean [SD]) increased 8.25 (47.39) μg/L from baseline in the 16-mg/day blonanserin group.

The incidence of AEs related to increased prolactin was 4.3% in the placebo, 15.7% in the 8-mg/day blonanserin, and 32.1% in the 16-mg/day blonanserin groups. The AE related to development comprised weight decreased in the placebo group (one patient) and weight increased in the 8-mg/day blonanserin group (two patients) (Table 6). Maximum weight increase of the two patients was observed at week 4 (4.2 and 6.2 kg), which although decreased at week 6 (2.0 and 5.2 kg). No clinically problematic changes in laboratory test values, vital signs, or body weight were observed (Table 8).

**Table 8.** Change from Baseline in Metabolic and Laboratory Parameters (Week 6, Last Observation Carried Forward)

	Placebo (*N* = 47)		Blonanserin (8 mg/day) (*N* = 51)		Blonanserin (16 mg/day) (*N* = 53)	
	n	*Mean (SD)*	n	*Mean (SD)*	n	*Mean (SD)*
Weight (kg)	47	−0.12 (2.001)	50	0.13 (2.329)	52	−0.33 (2.404)
*z*-Score of weight (kg)	47	−0.0132 (0.22656)	50	0.0124 (0.26274)	52	−0.0272 (0.26763)
Percentile of weight	47	−1.25 (8.384)	50	0.89 (8.899)	52	−0.62 (8.650)
Glucose (mg/dL)^a^	47	1.1 (7.98)	49	−1.1 (7.87)	52	0.2 (9.81)
Hemoglobin A1c (%)^a^	46	−0.03 (0.148)	49	0.01 (0.212)	52	−0.03 (0.155)
Triglycerides (mg/dL)^a^	47	−12.4 (38.59)	49	−0.1 (46.17)	52	−0.6 (38.74)
Total cholesterol (mg/dL)^a^	47	−2.8 (17.92)	49	−5.6 (26.47)	52	−2.9 (23.44)
Prolactin (μg/L)^a^						
Females	27	−9.398 (36.2509)	27	0.550 (28.6464)	29	8.246 (47.3911)
Males	20	−6.012 (23.2689)	20	−0.387 (17.7979)	23	−5.317 (36.5748)

^a^ Fasting conditions.

LOCF, last observation carried forward; SD, standard deviation.

## Discussion

A closed testing procedure was used in the order of step 1 and step 2 to adjust for test multiplicity in the analysis of the primary endpoint. The superiority of blonanserin over placebo was confirmed in step 1 (pooled blonanserin group). Subsequently, the superiority of blonanserin over placebo was verified for the 16-mg/day blonanserin group in step 2 (each dose group). The mean change in PANSS total score observed in the placebo and 16-mg/day blonanserin groups was −10.6 and −20.5, showing a moderate ES of 0.538, which was comparable with those reported for other atypical antipsychotics approved in the United States and European Union, etc. for adolescents with schizophrenia: mean reduction in placebo versus active (ES): olanzapine, −8.8 versus −21.3 (0.6); risperidone, −8.9 versus −21.2; quetiapine, −19.2 versus −28.4; paliperidone, −7.9 versus −17.3 (0.62); aripiprazole, −21.2 versus −28.6; and lurasidone, −10.5 versus −18.3 (0.51) (Findling et al. 2008, 2012; Haas et al. 2009; Kryzhanovskaya et al. 2009; Singh et al. 2011; Goldman et al. 2017). This result suggests that the efficacy of blonanserin is nearly similar to those second-generation antipsychotics for adolescents with schizophrenia.

Furthermore, from the result of the secondary endpoints, although multiplicity of statistical tests was not adjusted, oral administration of blonanserin tended to improve psychiatric symptoms. The percentage of PANSS responders in the blonanserin groups tended to be higher than that in the placebo group. At the improvement criteria of 30%–50%, a statistically significant difference was found between the 16-mg/day blonanserin and placebo groups. The change from baseline in CGI-S score tended to be high in the blonanserin groups compared with that in the placebo group. A statistically significant difference in CGI-I rate was found between the 16-mg/day blonanserin and placebo groups. Given these results, we consider that the sufficient improvement in psychiatric symptoms was achieved with oral administration of blonanserin in patients with adolescent schizophrenia.

Across all the PANSS subscale items, a larger reduction was observed in the blonanserin groups than in the placebo group. Moreover, in the PANSS 5-factor model scores, a larger reduction was observed with blonanserin than with placebo, suggesting that a comprehensive efficacy can be obtained with blonanserin. Remarkably, a larger reduction was observed in the blonanserin 16-mg/day group than in the placebo group, not only for positive score but also for negative and cognitive scores. With its efficacy for negative symptoms, which are common in the first episode of schizophrenia and adversely affect subsequent outcomes especially for adolescents (Correll and Schooler 2020), blonanserin may be a useful treatment option for adolescent schizophrenia.

Blonanserin improved verbal fluency and executive function (cognitive function) as well as daily living and work skills (social function) in adult patients with acute-phase schizophrenia (Hori et al. 2014). In *posthoc* analysis by MMRM in the present study, 16 mg/day blonanserin improved PANSS cognitive disorders score more than placebo. In addition, PANSS remission rate at week 6 (LOCF) was higher in 16-mg/day blonanserin group than in the placebo group, which might suggest that subsequent remission could be expected with blonanserin treatment compared with placebo similarly to that noted in adult schizophrenia (Ishigooka and Nakamura 2011). Taken together, blonanserin could possibly be a promising option in the therapeutic strategies aiming toward schoolwork or working in adolescents with schizophrenia.

The percentage of patients who developed any AE during the treatment phase was highest in the 16-mg/day blonanserin group, followed by the 8-mg/day blonanserin and the placebo groups. Similarly, withdrawal rate owing to AEs was highest in the 16-mg/day blonanserin group, followed by the 8-mg/day and placebo groups. No deaths and only a few serious/severe AEs were observed. The types and incidence rates of AEs were similar between adolescents in the present study and adults in previous studies on blonanserin (Murasaki 2007; Miura 2008; Garcia et al. 2009; Harvey et al. 2019, 2020). AEs caused by blonanserin in adolescent patients are considered as predictable as those in adult patients.

The most common AEs in the blonanserin groups (incidence ≥5%) can be classified as (1) a type with a high incidence compared with the placebo group, occurring dose dependently (extrapyramidal symptoms, somnolence, and increased prolactin level) or (2) a type with a similar incidence to the placebo group (nausea, headache, worsening schizophrenia, and skin abrasion). Because the incidence of the former increases with the increase in blonanserin dose, precautions for these events are needed when using blonanserin. However, because their occurrence may be predictable and discontinuation rate due to such events was 9.4% even in the 16-mg/day blonanserin group in the present study, these adverse effects are considered tolerable with sufficient monitoring, dosage adjustment, and concomitant medication use as necessary. In a randomized controlled study that compared blonanserin with risperidone in adult schizophrenia (Miura 2008), the incidence of akathisia and excitability was higher with blonanserin than with risperidone, whereas the incidence of prolactin increase, weight increase, and orthostatic hypotension was lower with blonanserin than with risperidone. Akathisia was also the AE with the highest incidence in the blonanserin groups in the present study.

Precautions for onset or exacerbation of akathisia similar to those in adults are necessary. In the present study, AEs related to excitability (excitability, irritability, hostility, aggression, and excitation) did not occur in any patients, and the reduction in excitement in the PANSS symptoms and 5-factor model was larger in the blonanserin groups than in the placebo group. Considering the low incidence of excitability, blonanserin can be safely indicated for adolescent schizophrenia, as long as sufficient precautions are taken against akathisia.

Early-onset schizophrenic adolescents are vulnerable to AEs, especially weight gain and metabolic side effects. Accordingly, the use of drugs with a high risk for weight gain as first-line agents should be limited (McClellan et al. 2013). In the present study, AEs related to weight decrease or increase were observed only in two with blonanserin treatment, and the mean weight change from baseline did not differ from that with placebo. Moreover, glucose tolerance or lipid metabolic parameters did not differ greatly from those with placebo. Several systematic reviews on antipsychotics for adolescent schizophrenia suggested a higher risk of weight increase with olanzapine, quetiapine, and risperidone, showing relatively low risk with aripiprazole among atypical antipsychotics (Harvey et al. 2016; Arango et al. 2020). In adult schizophrenia, blonanserin showed lower risk of weight increase than risperidone (Miura 2008).

Weight change observed with blonanserin in our study (8 mg/day 0.13 kg and 16 mg/day −0.33 kg at the end of the study) was similar to that observed with aripiprazole in a 6-week placebo-controlled study (10 mg/day 0.0 kg and 30 mg/day 0.2 kg at the end of the study) (Findling et al. 2008). These results might suggest that the risk of metabolic AEs, which are often associated with atypical antipsychotics and noted as the safety concern to be considered during drug selection in adolescent schizophrenia treatment guidelines worldwide, is presumed to be relatively low with blonanserin among second-generation antipsychotics. Therefore, in adolescent schizophrenia, the safety profile of blonanserin might be a useful treatment option in clinical practice.

The dose in the blonanserin groups was titrated up from an initial dose of 4 mg as a precautionary measure, and few patients, only one or two in each group, discontinued the treatment owing to progressive disease during the initial 2 weeks after study initiation. After 2 weeks of treatment, the number of patients who discontinued the treatment owing to AEs increased dose dependently. Based on these results, an appropriate dosage regimen of blonanserin for adolescence could be inferred as follows: the starting dose should be low, which should be gradually increased while monitoring its effectiveness and safety, finally reaching the highest dose of 16 mg/day at which the largest effect would be expected.

### Limitations

The results of this study were obtained in a short period using a limited group and methods. To further increase the generalizability of the evidence for drug therapy in adolescent schizophrenia, a study involving varied patient groups or actual clinical practice should be considered. In addition, further examination is required to determine the degree to which the efficacy and safety of blonanserin were affected by the higher percentage of concomitant use of psychotropics and hypnotics in the blonanserin groups than in the placebo group. Further examination is also needed to confirm the sustained efficacy and safety/tolerability during long-term administration.

## Conclusions

This study was the first Japanese placebo-controlled study involving adolescents with schizophrenia (12–18 years of age) and demonstrated that a 6-week dosing of oral blonanserin produced significant improvements in psychiatric symptoms over placebo in adolescents with schizophrenia. Safety profile of blonanserin in adolescents was similar to that in adults, with minimal effects on weight and metabolic parameters. Blonanserin may be a safe treatment option for adolescents with schizophrenia.

## Clinical Significance

Selection of atypical antipsychotic agents for pediatric and adolescent patients with schizophrenia requires consideration of the balance between effectiveness and safety, with particular focus on the safety profile. Blonanserin achieved a sufficient efficacy in adolescent patients and the safety profile was similar to that in adults, which suggests that blonanserin oral tablet is a beneficial drug treatment option for these patients.
